# Novel Financing Model for Neglected Tropical Diseases: Development Impact Bonds Applied to Sleeping Sickness and Rabies Control

**DOI:** 10.1371/journal.pntd.0005000

**Published:** 2016-11-17

**Authors:** Susan Christina Welburn, Kevin Louis Bardosh, Paul Gerard Coleman

**Affiliations:** 1 Division of Infection and Pathway Medicine, Edinburgh Medical School: Biomedical Sciences, College of Medicine and Veterinary Medicine, The University of Edinburgh, Chancellor’s Building, United Kingdom; 2 Department of Disease Control, Faculty of Infectious and Tropical Diseases, London School of Hygiene & Tropical Medicine, Keppel Street, Bloomsbury, London, United Kingdom; 3 H2O Venture Partners, United Kingdom; Swiss Tropical and Public Health Institute, SWITZERLAND

Sleeping sickness, or human African trypanosomiasis (HAT), epitomises the concept of a “neglected tropical disease” [1]. The history of HAT reads like a Hollywood parody of Homeric pestilence. Both the chronic and acute forms of HAT are exclusively confined to sub-Saharan Africa, where they affect the poorest of the rural poor—thriving in weak health systems and conflict zones—and where they have been responsible for massive historic epidemics [2]. Both forms of the disease are invariably fatal if not treated, but available therapies are difficult to administer, highly toxic, and increasingly pose problems of drug resistance [3]. Diagnostics are complex, and the procedures needed to sanction the more toxic treatments are difficult, painful, and dangerous, resulting in massive underreporting [4, 5]. The cunning ability of the causative parasites to evade the immune system has made a mockery of attempts to develop vaccines, and any continued protestations of a vaccine breakthrough on a meaningful timescale are, at best, wishful thinking [6]. Finally, the hope of control through targeting the disease vector, the tsetse fly, has (for the most part) eluded cost-effective, sustainable, large-scale delivery [7].

In the face of such challenges, the talk of effective disease control—elimination even—may at best be thought misplaced optimism and at worst detracting limited resources from a focus on further research to develop better tools for the future. However, disease elimination is a stated aim of the World Health Organization, and the moral imperative must be to deploy control tools to save lives now [8]. Successes arising from a small number of well-run programmes give confidence that even with existing tools, as limited as they may be, effective control—and in some areas elimination—is a realistic target [9]. The main challenge to realising these benefits is securing the necessary financing to enable appropriately designed interventions to be sustainably delivered at scale immediately [10].

HAT is caused by infection with one of two closely related but geographically discrete parasites that results in a progressive systemic disease followed by central nervous system damage and death. In Central and West Africa, disease progression resulting from *Trypanosoma brucei gambiense* infection is slow, occurring over many years. By contrast, in East Africa, *T*. *b*. *rhodesiense* infections progress rapidly, resulting in death within months of infection. While the total burden of disease is significantly greater for *T*. *b*. *gambiense* than *T*.*b*. *rhodesiense* HAT, where the latter occurs, local disease burden is much higher than might be expected from its relative incidence [11].

A major difference between the epidemiology of the two parasites is the importance of nonhuman animal hosts in the maintenance and spread of infection. As a good working approximation, *T*. *b*. *rhodesiense* is an infection of animals that occasionally spills over to humans [12], while *T*. *b*. *gambiense* is a human infection that occasionally resides in animals [13]. Through understanding this epidemiology, it is now possible to tailor existing tools to maximize benefit and deliver effective control.

This is what has been successfully done for controlling *T*. *b*. *rhodesiense* in Southeast and Central Uganda, where livestock (in particular, cattle) have been shown to be of central importance in the spread of sleeping sickness [14, 15]. Interventions targeted at controlling *T*. *b*. *rhodesiense* infections in livestock—mass administration of veterinary trypanocidal drugs followed up by the routine application of insecticides to protect cattle from tsetse flies—have been modelled [16, 17] and implemented across large areas of the endemic focus in Uganda following a resurgence of the disease in the early 2000s [18, 19].

These interventions deliver a double benefit. Firstly, they protect the human population from sleeping sickness and in so doing shift public health efforts to disease prevention rather than case finding and treatment. Secondly, they improve livestock health and productivity, as nonhuman infectious trypanosomes are a major veterinary health burden in sub-Saharan Africa that significantly affects the productivity of oxen and milking cows and hence rural livelihoods [20]. The societal impacts unlocked by delivering these interventions across the wider endemic areas of East Africa are potentially very large. The economic value that farmers place on their cattle provides a compelling private incentive to participate in disease control activities; indeed, it also opens up the potential to develop models for economic sustainability—overcoming the failures seen with tsetse control using traps—to incentivise and mobilise communities over the long term [21].

Ground-breaking work supported by long-term funding from the United Kingdom Department for International Development (DFID) has been undertaken in Uganda over the last two decades [14, 15, 16, 17, 18, 19]. The continued expansion of the area in Uganda affected by *T*. *b*. *rhodesiense*, threatening the integrity of the HAT focus for *T*. *b*. *gambiense* and risking overlap of the two forms of HAT, is a strong incentive for action [22]. The public–private alliance Stamp Out Sleeping sickness, (http://www.stampoutsleepingsickness.com/) delivered highly cost-effective control through mass cattle treatments and the development of innovative veterinary drug delivery networks. This prevented the spread of zoonotic HAT and has recently been named in the top 20 examples of UK further education institutions benefiting global development, selected from over 7,000 case studies (see: http://www.ukcds.org.uk/the-global-impact-of-uk-research/battling-sleeping-sickness). The WHO aims to eliminate sleeping sickness in Africa by 2030 [23]. The priority now is to scale these validated interventions and attract funding to allow sustained deployment across the entire region affected by HAT in Uganda.

**Fig 1:**
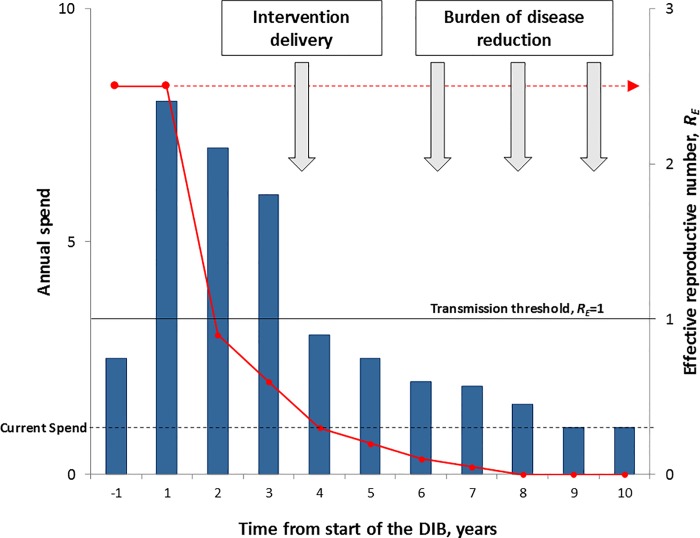
DIB cash flow and infectious disease control. The graph shows a stylised DIB cash flow profile (annual spend) and the impact on disease transmission (effective reproductive number, R_E_). Before the DIB is implemented, the current spend on control (broken black line) is inadequate to interrupt transmission (R_E_>1) and shows that in the absence of the additional financing, the disease would persist in an endemic state (broken red line). The DIB financing is shown by the blue bars and can be broken down into four phases. **Preimplementation Phase (Y–1)**: detailed design; establishment of surveillance systems; baseline surveys; piloting reporting systems; strengthening of the policy framework. **Suppression Phase (Y1–3)**: rollout of mass intervention campaigns; flexible, reactive management to achieve critical coverage and to interrupt transmission (R_E_<1); routine reporting; audit of intervention coverage. **Consolidation Phase (Y4–8)**: shift from mass intervention to surveillance and reactive interventions; protection against reintroduction of the disease. **Postcontrol Maintenance Phase (Y9–10)**: Capacity embedded in local system and ideally fully financed locally. The “front loaded” cash flow profile, which is characteristic of DIB financing, is ideally suited to infectious disease control and stands in contrast to the traditional flat year-on-year funding. Potential payment triggers for partial repayment of capital linked to intervention coverage and full capital repayments plus interest based on disease reduction are also shown (grey arrows).

To achieve this vision, DFID has funded research to explore a novel “impact investment” approach that could raise the necessary financing for sustained, scaled HAT control. The approach will build on, and benefit from, the success of GAVI-International Finance Facility for Immunisation (IFFIm) bonds and other social impact bond initiatives, whereby long-term public and/or donor commitments to support proven interventions are used to leverage upfront private sector investment. This financing approach, known as a Development Impact Bond (DIB), uses “private investment to provide upfront risk capital for development programmes, only calling on donor funding to repay capital, plus a potential return, once clearly defined and measured development outcomes are achieved” [24]. The ideal cash flow profile of the DIB (front-loaded investment followed by long-term lower cash needs) mirrors the high upfront effort needed to interrupt transmission followed by the reduced effort needed to maintain disease control. In poor countries, the money is rarely available to cover these upfront costs, and traditional donor grant funding does not advance large amounts of cash; in the DIB, private investors provide the capital needed upfront, at risk (see Fig 1).

Alongside the cash flow profile, the transfer of risk of programme failure from the donors to social investors is central to improving the likelihood of DIB financing over direct funding; DIBs could bring a greater focus on implementation and delivery of successful results. If the intervention does not work, the investors lose out. But if it succeeds, international donors repay the social investors with interest (at a rate below the market rate). In this way, DIBs can bring greater rigour to delivery of international development and global health interventions—investors will only back strategies that have evidence of success. The new instrument could promote greater scrutiny and operational flexibility to address the realities on the ground in running the campaign. Under the theoretical scenario outlined in Fig 1, the long-term maintenance of the disease-free state is embedded in local systems and ideally fully financed locally and affordable within the available budget deployed prior to control. The reality of this long-term sustainability is amongst many things that still need to be tested by the deployment of an actual DIB targeted against an NTD.

If a DIB for sleeping sickness can be developed and used to underpin successful disease control in Uganda, the financing approach could be extended to the other East African nations as well as the larger problem of *T*. *b*. *gambiense* sleeping sickness in Central and West Africa [13]. Of course, the systems developed through DIB financing to deliver existing interventions can be adapted to deliver improved control technologies (e.g., new therapeutics [25]) as the evidence base justifies their adoption. However, the broader applicability of DIBs could extend to the majority of the neglected tropical diseases, where imperfect but adequate disease control tools already exist.

Rabies is a prime example. The proposed interventions for zoonotic sleeping sickness control in Uganda share many properties of vaccination campaigns: the need for sensitisation of communities; the use of existing public health infrastructure capacity to allow routine delivery of proven interventions at scale; direct benefit(s) seen for the recipients of treatment; nonlinear benefits unlocked through increased coverage; a focus on prevention rather than reactive treatment; and highly cost-effective interventions and societal level benefits unlocked from deployment at scale, achieved without having to wait for the vaccine to be developed. However, we do have vaccines for rabies control—and have had for over 130 years! At the end of 2015, the WHO and the World Organization for Animal Health, in collaboration with the UN Food and Agriculture Organization and the Global Alliance for the Control of Rabies (GARC), launched a global framework to eliminate rabies by 2030 [26]. This is supported by the End Rabies Campaign launched by GARC in 2016 at the House of Lords in London, UK.

As with sleeping sickness, the constraints to rabies control are not technical but are rather the need for public engagement and the mobilization of the necessary financial resources to support effective operational delivery. The use of DIBs to achieve this, focused on mass vaccination of the domestic dog reservoir combined with the provision of postexposure therapy, is currently being explored.

Let us not retreat behind the promise of future success from continued research [10]; for many of the world’s neglected tropical diseases we already have the evidence and the infrastructure and, with DIBs, a potential financing model [27] for their elimination. With the new Global Goals for Sustainable Development in place, now is surely the time for action.
